# The Evolution and Current Landscape of Minimally Invasive Glaucoma Surgeries: A Review

**DOI:** 10.7759/cureus.52183

**Published:** 2024-01-12

**Authors:** Abdullah A Cheema, Haider R Cheema

**Affiliations:** 1 Ophthalmology, Royal Glamorgan Hospital, Cardiff, GBR; 2 Ophthalmology, Royal Berkshire Hospital, Reading, GBR

**Keywords:** diagnostic techniques < glaucoma preventive medicine/screening < socioeconomics and education in medicine/ophthalmology trauma socioeconomics and education in medicine/ophthalmology, complex glaucoma & cataract surgeries, glaucoma surgery, glaucoma treatment, primary open angle glaucoma, migs device, medical retina and glaucoma, glaucoma drainage device, minimally invasive glaucoma surgery

## Abstract

This review examines the evolution, current status, and future potential of minimally invasive glaucoma surgeries (MIGS), a significant advancement in the treatment of glaucoma, a leading cause of irreversible blindness. MIGS offer a less invasive alternative to traditional glaucoma surgeries, primarily aimed at reducing intraocular pressure, minimizing tissue trauma, and providing a safer profile. With the emergence of devices such as the Trabectome, iStent, and others, MIGS have expanded the surgical toolkit, allowing personalized, patient-centered care. Despite their advantages, MIGS face challenges such as efficacy in severe cases, long-term data, and accessibility. Ongoing research and technological innovations continue to refine their capabilities and applications, promising to further transform glaucoma management and patient outcomes. This paper provides an in-depth analysis of MIGS, reflecting on their impact and contemplating future directions in this dynamically evolving field.

## Introduction and background

Glaucoma, a leading cause of irreversible blindness globally, is a complex group of diseases primarily characterized by the progressive degeneration of the optic nerve, often associated with elevated intraocular pressure (IOP) [1]. The global burden of glaucoma is significant, affecting millions of individuals, and is projected to increase as the population ages.

The pathophysiology of glaucoma is multifaceted, involving mechanical and vascular factors that lead to optic nerve damage and visual field loss [2]. Elevated IOP is recognized as the most significant modifiable risk factor. While the initial management of glaucoma often involves pharmacological treatments and laser therapy, these approaches may not be sufficient for all patients. In cases where medication and laser therapies are inadequate in controlling IOP or when they lead to unacceptable side effects, surgical intervention becomes necessary.

Traditional surgical options for glaucoma, such as trabeculectomy and implantation of glaucoma drainage devices (GDDs) (e.g., Ahmed, Baerveldt, and Molteno implants), have been the mainstay for decades. These procedures, while effective in reducing IOP, are associated with potential complications, including infection, hypotony, suprachoroidal hemorrhage, and cataract formation [3]. Moreover, they often involve extensive surgical dissections and postoperative care, which can be challenging for both the patient and the healthcare system.

Minimally invasive glaucoma surgeries (MIGS) offer a less invasive alternative, with the primary goals of reducing IOP, minimizing tissue trauma, and offering a better safety profile compared to traditional glaucoma surgeries [4]. The term MIGS encompasses a variety of procedures that share common characteristics: they are generally performed through small incisions, cause minimal trauma to the target tissues, and have a favorable safety profile.

MIGS can be broadly categorized into three distinct types [5]. The first category comprises surgeries that focus on increasing trabecular outflow. This is achieved through the use of innovative devices, including Trabectome, iStent (available in both first and second generations), Hydrus microstent, Kahook Dual Blade (KDB), and gonioscopy-assisted transluminal trabeculotomy. These devices are designed to enhance the natural drainage pathways for aqueous humor, thereby reducing IOP, a key factor in glaucoma management [5].

The second category involves surgeries that aim to enhance suprachoroidal outflow. This is facilitated by devices such as the Cypass Microstent and iStent Supra. These surgeries target a different drainage pathway in the eye, offering an alternative approach to managing IOP [5].

Lastly, the third category includes conjunctival bleb-forming procedures, exemplified by the use of the XEN Gel Stent and InnFocus Microshunt. These procedures create a new drainage pathway for the aqueous humor, thereby reducing IOP by diverting the fluid to an area where it can be absorbed [5].

The development of MIGS represents a significant advance in glaucoma management, aiming to fill the gap between conservative management and more invasive surgical options [5]. This review article seeks to explore the evolution, current state, and future potential of MIGS, providing an in-depth analysis of their role in the contemporary glaucoma treatment landscape.

Historical context

Prior to the advent of MIGS, the landscape of glaucoma surgery was dominated by traditional procedures that were often characterized by their invasiveness and the higher risk of complications [6]. Understanding this historical context is crucial to appreciate the significance of MIGS in the evolution of glaucoma treatment.

Traditional Surgical Approaches

Introduced in the 1960s, trabeculectomy has been a cornerstone in glaucoma surgery for decades and remains the gold standard for drainage procedures. This procedure involves creating a partial-thickness flap in the sclera, followed by the excision of a piece of the underlying trabecular meshwork and Schlemm's canal to form a new outflow pathway for the aqueous humor into the subconjunctival space, thereby reducing IOP. Despite its effectiveness in lowering IOP, trabeculectomy is associated with significant risks such as hypotony (abnormally low IOP), infection, and failure due to scar formation [7,8].

GDDs, such as the Ahmed, Baerveldt, and Molteno implants, were introduced to manage cases where trabeculectomy was less likely to succeed, particularly in patients with neovascular glaucoma, uveitic glaucoma, or in those with previous conjunctival scarring. These devices create an alternative pathway for aqueous humor to flow from the anterior chamber to an external reservoir, thereby lowering IOP. While effective, they carry risks of complications such as tube exposure, erosion, blockage, and migration, as well as the potential for postoperative hypotony [7].

Cycloablative procedures such as cyclocryotherapy and cyclophotocoagulation were developed to reduce aqueous humor production by ablating the ciliary body. These were typically reserved for advanced, refractory glaucoma cases or when other surgeries were not feasible. The risk of serious complications, including phthisis (shrinkage of the eyeball), chronic inflammation, and loss of vision, limited their use [7].

Deep sclerectomy and viscocanalostomy were developed as non-penetrating surgical alternatives to trabeculectomy, these techniques aim to reduce IOP by improving the outflow of aqueous humor without creating a full-thickness opening into the anterior chamber. These procedures are technically challenging and have not gained widespread acceptance due to variable success rates and the need for specialized training [6].

Limitations of Traditional Surgeries

The traditional surgical approaches, while effective in lowering IOP, were often associated with a significant risk of both intraoperative and postoperative complications [7]. These procedures typically required extensive postoperative care, including frequent follow-up visits and adjustments. The risk of sight-threatening complications, coupled with the need for prolonged healing time, often made these options less desirable for patients with mild to moderate glaucoma [7].

Additionally, the success of these surgeries was heavily dependent on the surgeon's expertise and the patient's individual response to surgery, which added a level of unpredictability to the outcomes [8]. The potential for serious complications and the invasive nature of these procedures created a need for a safer, less invasive surgical option, paving the way for the development of MIGS.

## Review

Genesis of MIGS

Early Developments

The inception of MIGS marks a significant turning point in the field of glaucoma management [9]. The early developments of MIGS were driven by the desire to provide a safer, less invasive alternative to traditional glaucoma surgeries while effectively managing IOP.

The fundamental concept behind MIGS is to offer a surgical option that bridges the gap between conservative management (medications and laser treatments) and the more invasive traditional glaucoma surgeries [9]. The initial idea was to develop procedures that could be performed through small incisions, causing minimal trauma, with a rapid postoperative recovery and a more favorable safety profile.

The evolution of MIGS began with the development of devices designed to bypass the primary site of aqueous humor outflow resistance in open-angle glaucoma. This resistance is typically located at the trabecular meshwork and Schlemm’s canal. The early MIGS devices focused on enhancing aqueous outflow through these structures.

Approved by the FDA in 2004, Trabectome (Neomedix Corporation, Tustin, CA) was one of the first MIGS devices [10]. It works by ablating a portion of the trabecular meshwork and the inner wall of Schlemm’s canal to enhance aqueous outflow, thereby lowering IOP. It was a pioneering device in demonstrating the potential of MIGS to offer a safer, more effective IOP-lowering intervention.

Another early development in the MIGS field was iStent (Glaukos Corporation, Laguna Hills, CA), which received FDA approval in 2012 for use in conjunction with cataract surgery. iStent is a tiny bypass device implanted into Schlemm’s canal to facilitate aqueous outflow [11]. Its introduction represented a significant step forward in the MIGS arena, offering a minimally traumatic approach with the potential for substantial IOP reduction.

These initial devices set the stage for a rapidly expanding field of glaucoma surgery. They were designed to be less traumatic, with the goal of reducing the risk of complications associated with traditional glaucoma surgeries. By focusing on enhancing the natural aqueous outflow pathways, these first-generation MIGS devices sought to offer a balance between efficacy and safety.

The introduction of these early MIGS devices offered glaucoma surgeons and their patients new options, particularly for those with mild to moderate disease or those who were at higher risk for complications from traditional surgeries [8]. The success of these first-generation MIGS devices also spurred further innovation and development in the field, leading to the introduction of a variety of new techniques and devices designed to address different aspects of aqueous humor dynamics.

Technological advancements in MIGS

The evolution of MIGS has been significantly propelled by key technological innovations. These advancements not only improved the efficacy and safety of these procedures but also broadened the scope of their applicability in glaucoma management [10].

Micro-surgical Equipment and Techniques

One of the fundamental advancements in the development of MIGS was the adoption of micro-incisional surgical techniques [12]. This approach minimizes tissue trauma and hastens postoperative recovery. The use of specialized micro-surgical instruments allowed for precise interventions with reduced surgical footprint.

The advent of advanced microscopes and intraoperative imaging technologies, such as high-definition gonioscopy and anterior segment optical coherence tomography (OCT), provided surgeons with enhanced visualization of the angle structures. This improvement was critical for the precise placement of MIGS devices and for ensuring the safety and success of the procedures.

Device Innovations

Significant advancements were made in the design and materials of stents and scaffolds used in MIGS [13]. These devices were engineered to be biocompatible, non-erodible, and conducive to natural aqueous outflow [13]. The introduction of various stents, such as iStent, Hydrus microstent, and others, exemplifies this innovation. Each design addressed specific aspects of aqueous humor dynamics, from bypassing the trabecular meshwork to scaffolding Schlemm’s canal to prevent collapse.

The development of devices such as the Trabectome and KDB revolutionized trabecular meshwork ablation [14]. These devices allowed for targeted removal of trabecular tissues, enhancing aqueous humor outflow while preserving the structural integrity of the eye.

Integration of Diagnostic and Surgical Planning Tools

The integration of advanced diagnostic tools into preoperative planning significantly enhanced the precision and customization of MIGS [12,15]. Technologies such as OCT provide detailed imaging of the anterior segment, enabling surgeons to assess the angle anatomy and plan the surgical approach more accurately.

Real-time monitoring technologies also played a pivotal role in ensuring the accuracy and safety of MIGS [15]. The ability to monitor IOP changes during the procedure allowed for immediate adjustments, ensuring optimal outcomes [16].

Impact of Technological Advancements on MIGS

These technological innovations greatly expanded the capabilities and safety profile of MIGS. By enabling surgeons to perform more precise and less invasive procedures, these advancements have significantly improved patient outcomes [12,13]. The reduced risk profile and quicker recovery associated with MIGS have made them an attractive option for a broader range of glaucoma patients, including those with mild to moderate disease severity.

Milestones in MIGS development

The journey of MIGS through various stages of development, clinical trials, and FDA approvals has marked significant milestones in the field of glaucoma treatment. These milestones reflect the evolving understanding of glaucoma pathophysiology and the pursuit of safer, effective surgical interventions [16].

FDA Approvals

A pioneering device in the MIGS category, Trabectome (Neomedix Corporation, Tustin, CA) received FDA approval in April 2004 [4]. It represented a significant advancement in angle-based surgeries, targeting the trabecular meshwork for enhanced aqueous outflow. Glaukos Corporation’s iStent, the first-generation micro-bypass stent, was approved by the FDA in June 2012. It gained recognition for its role in reducing IOP when combined with cataract surgery, offering a new approach for patients undergoing concurrent procedures [15].

The FDA approval of Ivantis Inc.'s Hydrus Microstent in 2018 was another landmark in the MIGS landscape [11]. This device, designed to scaffold Schlemm’s canal, demonstrated significant IOP reduction in clinical trials. This was preceded by approval by the FDA in 2016 of the XEN Gel Stent (Allergan, Dublin, Ireland) and provided an alternative approach by creating a subconjunctival drainage pathway, diverging from traditional angle-based MIGS procedures [17]. Lastly, registered with the FDA in 2015, the KDB (New World Medical, Rancho Cucamonga, CA) offered a novel approach to trabecular meshwork ablation, further diversifying the MIGS options [17].

Landmark Studies

The landmark studies for various MIGS devices have played a crucial role in their FDA approvals and clinical adoption. The iStent clinical trials were pivotal for the first-generation iStent, demonstrating its efficacy in reducing IOP, particularly when used alongside cataract surgery, thus establishing its role in managing mild to moderate glaucoma [15,17]. The Hydrus Microstent's approval was bolstered by comprehensive clinical trials, including the HORIZON study, which evidenced significant IOP reduction and decreased need for medications compared to just cataract surgery [13]. For the XEN Gel Stent, extensive clinical evaluations highlighted its effectiveness in lowering IOP and reducing medication use, especially in patients for whom traditional surgeries posed greater risks [13].

These FDA approvals and the supportive evidence from landmark studies have not only validated the safety and efficacy of MIGS but also paved the way for their broader acceptance globally. Ophthalmologists worldwide have increasingly incorporated MIGS into their glaucoma treatment algorithms, particularly for patients seeking less invasive options or those with mild to moderate disease severity [12,13].

The milestones in MIGS development represent a significant shift towards patient-centered, minimally invasive glaucoma care, offering a blend of safety, efficacy, and quicker recovery times.

Global acceptance of MIGS

The acceptance and adoption of MIGS have seen a significant rise across various regions of the world, profoundly impacting global glaucoma management practices. This global embracement is a testament to the growing recognition of the benefits MIGS in terms of safety, efficacy, and patient satisfaction [18].

Adoption in Different Regions

The United States has been at the forefront of MIGS development and adoption, primarily due to early FDA approvals and extensive clinical research [19]. The integration of MIGS into the treatment paradigm was facilitated by comprehensive training programs for surgeons and the establishment of MIGS as a standard care option in many institutions.

European countries have also shown significant interest in MIGS, with many adopting these procedures soon after their respective regulatory approvals [13]. European glaucoma specialists have been instrumental in conducting clinical trials and studies that further substantiate the role of MIGS in glaucoma management.

In the Asia-Pacific region and other emerging markets [19], the adoption of MIGS has been steadily increasing. Factors such as rising healthcare standards, increasing awareness of glaucoma treatments, and the prevalence of glaucoma in these regions have contributed to this trend.

Impact on Glaucoma Management Practices

Shift in surgical approach: MIGS have caused a shift in surgical glaucoma management, particularly for patients with mild to moderate glaucoma. Surgeons now have the option to offer a less invasive surgical intervention earlier in the disease course, potentially altering the natural progression of glaucoma.

Enhanced patient acceptance and satisfaction: The reduced invasiveness and lower risk profile of MIGS have led to higher patient acceptance and satisfaction [20]. This shift is particularly evident in patients who are apprehensive about traditional glaucoma surgeries due to their associated risks and longer recovery times.

Training and education: The global acceptance of MIGS has necessitated the incorporation of these procedures into ophthalmic training programs [21]. As a result, there has been an increased emphasis on educating and training new and established glaucoma surgeons in MIGS techniques [21].

Economic considerations: The adoption of MIGS has also been influenced by economic factors, such as cost-effectiveness, reimbursement policies, and healthcare infrastructure [22]. In regions where healthcare resources are limited, MIGS offer an attractive alternative due to their shorter operative times and reduced need for postoperative care [22].

The global acceptance of MIGS is a reflection of the ongoing evolution in glaucoma care, emphasizing patient safety, efficacy, and quality of life. As MIGS continue to evolve with new devices and techniques, their role in global glaucoma management is likely to expand further, offering promising avenues for enhanced patient care [12].

Current techniques and devices in MIGS

The landscape of MIGS has expanded considerably, encompassing a variety of techniques and devices [13]. Each of these approaches is designed to reduce IOP through different mechanisms of action, catering to the specific needs of various glaucoma patients.

Overview of MIGS Techniques

Trabecular meshwork bypass: This approach involves bypassing the trabecular meshwork, the primary resistance site in open-angle glaucoma, to create a direct pathway for aqueous humor into Schlemm's canal [10,23]. Devices such as iStent and the newer iStent inject are implanted into Schlemm's canal to facilitate outflow, with the latter allowing multiple stents. The Hydrus Microstent, a longer stent, scaffolds a segment of Schlemm's canal to enhance outflow across a broader area. These devices have been effective in reducing IOP, especially when used in conjunction with cataract surgery [15].

Suprachoroidal shunts: Targeting the uveoscleral pathway, this technique involves placing a shunt in the suprachoroidal space to enhance aqueous humor flow into this area [11,24]. The Cypass Micro-Stent was designed for this purpose but was withdrawn from the market due to concerns about long-term endothelial cell loss. The long-term efficacy of suprachoroidal shunts is still under evaluation.

Subconjunctival filtration: This method creates a new drainage pathway from the anterior chamber to the subconjunctival space, forming a filtration bleb [12]. The XEN Gel Stent, made from soft, collagen-derived gelatin, effectively creates a subconjunctival outflow pathway. It has been effective in lowering IOP and reducing glaucoma medication needs, with a safety profile comparable to traditional filtration surgeries [25].

Trabecular meshwork ablation: Involving the selective removal of trabecular meshwork tissue to enhance aqueous outflow [10,13], this technique uses devices such as the KDB. The KDB, designed for trabecular meshwork tissue excision, improves outflow through Schlemm’s canal and has shown efficacy in reducing IOP with a favorable safety profile [26]. This is summarized in Table 1.

**Table 1:** Overview of MIGS Devices and Their Mechanisms IOP: Intraocular Pressure Sources: [13-17]

MIGS Technique	Device	Mechanism of Action	Clinical Efficacy
Trabecular Meshwork Bypass	iStent, iStent inject, Hydrus Microstent	Bypass trabecular meshwork to improve outflow into Schlemm’s canal	Effective IOP reduction, especially in conjunction with cataract surgery
Suprachoroidal Shunts	Cypass Micro-Stent (withdrawn)	Enhance uveoscleral outflow	Promising initial results, long-term data under evaluation
Subconjunctival Filtration	XEN Gel Stent	Create new drainage pathway to subconjunctival space	Comparable to traditional filtration surgeries in IOP reduction
Trabecular Meshwork Ablation	Kahook Dual Blade	Selective removal of trabecular meshwork	Effective in reducing IOP with a favorable safety profile

The current array of MIGS techniques and devices offers glaucoma surgeons a diversified toolkit for personalized patient care [21]. While each technique has its unique mechanism and indications, they all share the common goal of minimizing surgical trauma and maximizing patient outcomes. As MIGS continues to evolve, further research and long-term studies will likely expand their application and efficacy in glaucoma management.

Specific Devices in MIGS

In the realm of MIGS, several devices have risen to prominence, each with its unique features and specific indications.

iStent and iStent Inject (Glaukos Corporation, Aliso Viejo, CA): The iStent is a small L-shaped stent designed to bypass the trabecular meshwork into Schlemm's canal, while the iStent Inject system facilitates the implantation of two stents for enhanced aqueous humor outflow [14,23]. Notably, iStent is one of the smallest medical devices implanted in the human body. Its primary clinical application is in conjunction with cataract surgery for patients with mild to moderate open-angle glaucoma [12].

Hydrus Microstent (Ivantis, Inc., Irvine, CA): This 8-mm long, crescent-shaped device scaffolds a portion of Schlemm’s canal, offering a broader area of outflow than traditional single-point stents [16,24]. Made of super-elastic, biocompatible nitinol, the Hydrus Microstent is indicated for use alongside cataract surgery in patients with mild-to-moderate open-angle glaucoma.

XEN Gel Stent (Allergan, Dublin, Ireland): Creating a subconjunctival drainage pathway to reduce IOP, this soft, collagen-derived gelatin tube allows aqueous humor to flow from the anterior chamber to the subconjunctival space [11,24]. The stent is about 6 mm long and highly flexible, adopting a minimally invasive approach similar to traditional trabeculectomy. It is particularly suitable for patients requiring significant IOP reduction where traditional surgery is high-risk [16].

KDB (New World Medical, Rancho Cucamonga, CA): Designed for the excision of trabecular meshwork tissue to improve outflow through Schlemm’s canal, the KDB's dual blade design enables precise targeting and removal of trabecular meshwork with minimal collateral damage [9,18,22]. It is used in patients with open-angle glaucoma, particularly those not well-managed with topical medications or where other MIGS options are not suitable [16]. This is summarized in Table 2.

**Table 2:** Summary of Common MIGS Devices IOP: Intraocular Pressure, MIGS: Minimally Invasive Glaucoma Surgeries Sources: [9-13]

Device	Manufacturer	Mechanism of Action	Unique Features	Clinical Application
iStent/iStent Inject	Glaukos Corporation	Bypass trabecular meshwork to Schlemm's canal	One of the smallest implanted devices; allows multiple stent implantation	Used with cataract surgery for mild to moderate glaucoma
Hydrus Microstent	Ivantis Inc.	Scaffold Schlemm’s canal	Long, crescent-shaped design for broad canal dilation	Used with cataract surgery for mild to moderate glaucoma
XEN Gel Stent	Allergan	Create subconjunctival drainage pathway	Flexible, collagen-derived tube for minimal trauma	Suitable for significant IOP reduction, high-risk cases
Kahook Dual Blade	New World Medical	Excise trabecular meshwork	Dual blade for precise trabecular meshwork removal	For patients not managed by medications or other MIGS

The diversity and specificity of these MIGS devices have greatly expanded the surgical options available for glaucoma management. By offering different mechanisms of action and unique features, these devices allow for tailored treatments to meet individual patient needs and disease severities [26].

Efficacy profile of MIGS

The efficacy and safety profiles of MIGS have been a focal point in their clinical assessment. Numerous studies and trials have documented their effectiveness in reducing IOP and managing glaucoma, alongside their safety profiles [27].

Variability in IOP reduction: The IOP reduction achieved by different MIGS procedures shows considerable variability. The highest IOP reduction was observed with the XEN Gel Stent (median of 11.0 mm Hg) and Micropulse CPC (16.5 mmHg for MCPC). In contrast, less pronounced reductions were noted in procedures such as KDB and Micropulse Diode Laser Trabeculoplasty (MDLT), with reductions of 2.4 mm Hg and 2.5 mm Hg, respectively [28,29].

Comparative effectiveness: Some studies compared the effectiveness of MIGS with other treatments. For instance, in the case of CyPass Micro-Stent and Hydrus Microstent, both showed greater IOP reduction compared to control groups undergoing phacoemulsification only [23,25].

Impact of multiple devices: In procedures where multiple devices were used, such as iStent (Multiple Stents), there was a trend of increased IOP reduction with the number of stents implanted, ranging from 7.6 mm Hg with one stent to 10.9 mm Hg with three stents [22].

Topical medication reduction: Most MIGS procedures also resulted in a significant reduction in the need for topical medications. For example, the CyPass Micro-Stent showed a reduction of 1.2 medications, and the Hydrus Microstent saw a reduction of 1.4 medications, indicating a substantial decrease in medication burden post-surgery [23,27].

Success rates: The studies varied in how they defined success, often considering both IOP reduction and reduction in medication use. For example, 76% of phaco-iStent eyes achieved an unmedicated IOP reduction of ≥20% at two years [23]. The follow-up periods in these studies ranged from three months to 42 months, indicating the need for long-term data to fully understand the durability of these procedures.

These findings, summarized in Table 3, highlight the diverse efficacy of MIGS procedures in terms of IOP reduction and medication burden. They underscore the importance of selecting the appropriate MIGS procedure based on individual patient profiles and the specific outcomes desired, such as the degree of IOP reduction or medication reduction [13]. This variability also emphasizes the need for further long-term studies to better understand the lasting impacts of these procedures [19].

**Table 3:** Summary the Efficacy Data of Various Minimally Invasive Glaucoma Surgeries ABiC: Ab-Interno Canalopasty, MCPC: Micropulse Cyclophotocoagulation, CWCPC: Continuous-Wave Cyclophotocoagulation, ELT: Excimer Laser Trabeculotomy, SLT: Selective Laser Trabeculoplasty, KDB: Kahook Dual Blade, MDLT: Micropulse Diode Laser Trabeculoplasty, ALT: Argon Laser Trabeculoplasty Sources: [13-19,21,23,25,29]

Procedure	IOP Reduction (mm Hg)	Topical Medication Reduction
Ab-Interno Canaloplasty (ABiC)	9	1.96
Micropulse CPC	16.5 (MCPC) vs. 16 (CWCPC)	1
CyPass Micro-Stent	7.4 (phaco-CyPass) vs. 5.4 (phaco-only)	1.2 vs. 0.7
Excimer Laser Trabeculotomy (ELT)	7.4 (ELT) vs. 4.8 (SLT)	1.54 vs. 1.33
Hydrus Microstent	7.6 (phaco-Hydrus) vs. 5.3 (phaco-only)	1.4 vs. 1.0
iStent and iStent Inject	7.0 (phaco-iStent) vs. 5.4 (phaco-only)	1.2 vs. 0.8
iStent (Multiple Stents)	7.6 (one stent) vs. 9.2 (two stents) vs. 10.9 (three stents)	Unavailable
iStent (Comparison with Travoprost)	10.9 (iStent) vs. 9.8 (travoprost)	-0.2 vs. -0.3 classes
Kahook Dual Blade (KDB)	2.4 (phaco-KDB) vs. 1.7 (phaco-iStent)	0.6 vs. 1.3
Micropulse Diode Laser Trabeculoplasty (MDLT)	2.5 (MDLT) vs. 4.9 (ALT)	0.3 vs. -0.1
Trabectome	6.6 (phaco-Trabectome) vs. 2.5 (phaco-only)	-0.1 vs. 0.1
XEN Gel Stent	Median 11.0 (both groups)	Median 3.0 (both groups)

Safety profile of MIGS

While MIGS have shown significant efficacy in reducing IOP, understanding their safety profile and potential complications is crucial for comprehensive glaucoma management. The safety considerations of MIGS are a key component in their growing acceptance among ophthalmologists and patients [25,28].

Potential Complications Associated with Specific MIGS Devices

Common complications with the iStent and iStent Inject include transient hyphema and IOP spikes. Other less frequent issues can involve stent obstruction or malposition [12]. These complications are usually manageable with conservative treatment or minor interventions [30].

Similar to the iStent, the Hydrus Microstent may cause transient hyphema and IOP spikes. Complications related to the insertion process, such as trauma to the trabecular meshwork or Schlemm’s canal, are also possible [31]. Most of these complications are transient and can be effectively managed postoperatively.

The XEN Gel Stent device can lead to bleb-related issues, such as bleb fibrosis or failure, the device slipping into the anterior chamber, and, less commonly, infection or hypotony [31]. These complications might require additional interventions, such as bleb needling or revision, and are managed similarly to traditional filtering surgery complications.

The KDB may cause transient intraoperative bleeding and IOP fluctuations. Damage to adjacent ocular structures, although rare, is also a potential complication. Generally, these issues are manageable with standard postoperative care and seldom require further surgical intervention [25].

The safety profile of MIGS is generally favorable, with most complications being mild and manageable. The reduced risk of serious complications compared to traditional surgeries makes MIGS an attractive option for many patients, particularly those with less advanced glaucoma. However, it is crucial for clinicians to be aware of the potential complications and have strategies in place for their management. This is summarized in Table 4.

**Table 4:** Safety Profile and Complications in MIGS IOP: Intraocular Pressure Sources: [21-29]

Device	Common Complications	Management Strategies
iStent/iStent Inject	Transient hyphema, IOP spikes	Conservative treatment, minor interventions
Hydrus Microstent	Transient hyphema, IOP spikes, insertion trauma	Postoperative management, conservative treatment
XEN Gel Stent	Bleb-related issues, infection, hypotony	Additional interventions, similar to filtering surgery
Kahook Dual Blade	Intraoperative bleeding, IOP fluctuations	Standard postoperative care

Complication Rates

Overall complication rate: Across 16 of 20 studies analyzed by Nichani et al. [23], a total of 1,207 complications were identified in 3,199 eyes (37.7%) that underwent a MIGS intervention. This included 738 complications (45.0%), among 1,639 eyes with a phaco-MIGS procedure, 268 complications (42.3%) in 633 phaco-only eyes, 124 complications (21.9%) in 566 standalone-MIGS eyes, and 77 complications (21.3%) in 361 eyes without one of the aforementioned procedures [31]. The most common complications included stent obstruction (14.5% of MIGS complications), inflammation (13.5% in MIGS vs. 16.7% in non-MIGS), surgical reintervention (10.7% vs. 13.0%), and decreased visual field (5.1% vs. 7.7%). Ocular surface disease complicated 16.1% of phaco-MIGS and 16.8% of phaco-only eyes [23,24,26]. This is summarized in Table 5.

**Table 5:** Complication Rates in MIGS Procedures Sources: [23,24,26]

Procedure	Complications	Common Complications
Phaco-MIGS	738 (45.0%)	Stent obstruction (14.5%), Inflammation (13.5%), Surgical reintervention (10.7%), Decreased visual field (5.1%)
Phaco-Only	268 (42.3%)	Inflammation (16.7%), Ocular surface disease (16.8%)
Standalone-MIGS	124 (21.9%)	Unspecified
Other	77 (21.3%)	Unspecified

Reoperation Rates

Phaco-Hydrus vs. Cataract surgery alone: The HORIZON study showed surgical reintervention rates of 3.5% for phaco-Hydrus vs. 7.5% for cataract surgery alone at 24 months. Another study reported reoperation rates of 2.1% vs. 4.1% at 24 months [29,30].

Phaco-CyPass vs. Cataract surgery alone: The COMPASS study indicated reoperation rates of 5.5% for phaco-CyPass vs. 5.3% for cataract surgery alone at 24 months.

Phaco-iStent vs. Cataract surgery alone: One study showed reoperation rates of 2.1% for phaco-iStent vs. 4.1% for cataract surgery alone at 12 months. Another study comparing phaco-iStent (G2) to cataract surgery alone reported reoperation rates of 5.4% vs. 5.0% at 24 months [23,30]. This is summarized in Table 6.

**Table 6:** Reoperation Rates in MIGS Procedures Sources: [23,25,29,30]

Procedure Comparison	Reoperation Rates
Phaco-Hydrus vs. Cataract Surgery	3.5% vs. 7.5%
Phaco-CyPass vs. Cataract Surgery	5.5% vs. 5.3%
Phaco-iStent vs. Cataract Surgery	2.1%-5.4% vs. 4.1%-5.0%

Postoperative Care

The analysis [23] revealed that 41.2 events occurred per 100 eyes after a MIGS intervention (standalone or in combination with another intervention), most of which resolved within a week to a month and were not serious, visually threatening, or clinically significant [31]. The clinical outcomes of MIGS are summarized in Table 7.

**Table 7:** Summary of Clinical Outcomes in MIGS IOP: Intraocular Pressure Sources: [23,25,27,30]

Device	Efficacy in IOP Reduction	Safety Profile
iStent/iStent Inject	Effective, especially with cataract surgery	Low incidence of serious complications
Hydrus Microstent	Significant IOP control in conjunction with cataract surgery	Comparable to cataract surgery alone
XEN Gel Stent	Effective in reducing IOP and medication burden	Comparable to traditional filtering surgeries
Kahook Dual Blade	Significant IOP reduction	Minimal intraoperative and postoperative complications

MIGS have demonstrated significant efficacy in reducing IOP and managing glaucoma, with a safety profile that is generally more favorable than traditional glaucoma surgeries. Their minimally invasive nature, combined with effective outcomes, makes them a valuable option in the treatment of glaucoma, particularly for patients with mild to moderate disease severity.

Comparison with traditional surgeries

MIGS have emerged as a significant advancement in the field of glaucoma treatment, offering distinct advantages over traditional surgical approaches. These advantages are central to understanding the increasing preference for MIGS in managing glaucoma, particularly in patients with mild to moderate disease severity [30].

Advantages of Traditional Approaches 

Reduced complication rates: Traditional glaucoma surgeries, such as trabeculectomy and GDDs, carry a higher risk of complications such as hypotony, infection, and suprachoroidal hemorrhage [27]. MIGS, with their minimally invasive nature, significantly reduce these risks. The smaller incisions and less invasive techniques result in fewer instances of severe complications [27].

Shorter recovery time: Recovery from traditional surgeries can be prolonged and demanding, often requiring extensive postoperative care. MIGS patients typically experience a quicker recovery period, allowing for a faster return to daily activities and less postoperative discomfort [30].

Lower postoperative care burden: Postoperative management of traditional surgeries often involves frequent follow-ups and intensive care, including adjustments and management of complications [9,23]. MIGS, with their lower complication rates, require less intensive postoperative care, reducing the burden on both patients and healthcare systems [23].

Potential for earlier intervention: Traditional surgeries are often reserved for advanced or uncontrolled cases of glaucoma due to their risk profiles. The safety profile of MIGS allows for their consideration earlier in the disease course, potentially altering disease progression and outcome [23].

Quality of life considerations: The invasiveness and complication risks of traditional surgeries can impact the patient's quality of life. MIGS offer an effective treatment with a lower impact on quality of life, making them a more favorable option for many patients [31]. This is summarized in Table 8.

**Table 8:** MIGS vs. Traditional Surgeries Sources: [15,17,23,27-30]

Aspect	Traditional Surgeries	MIGS
Complication Rate	Higher (e.g., hypotony, infection)	Significantly lower
Recovery Time	Longer, more demanding	Shorter, less discomfort
Postoperative Care	Intensive, frequent follow-ups	Less intensive, fewer follow-ups
Timing of Intervention	Often in advanced stages	Possible earlier in disease
Quality of Life Impact	Potentially significant	Generally lower

MIGS have redefined the surgical management of glaucoma by offering a safer, more patient-friendly approach compared to traditional surgeries. Their reduced complication rates, shorter recovery times, and the potential for earlier intervention make them an increasingly popular choice in glaucoma treatment strategies [32]. This shift reflects a broader trend in medicine towards procedures that maximize efficacy while minimizing invasiveness and patient burden [33].

Limitations of MIGS

While MIGS represent a significant advancement in glaucoma treatment, they are not without limitations and challenges. A comprehensive understanding of these aspects is crucial for clinicians in making informed treatment decisions. This section outlines the current limitations and challenges faced by MIGS in comparison to traditional glaucoma surgeries.

Efficacy in severe glaucoma: MIGS are primarily effective in mild to moderate glaucoma. Their capacity for substantial IOP reduction in severe cases is less significant compared to traditional surgeries such as trabeculectomy or GDDs [23]. Consequently, patients with advanced glaucoma requiring significant IOP reduction may not be ideal candidates for MIGS.

Long-term efficacy data: There is a notable lack of long-term data on the sustainability of IOP control with MIGS. While most studies report shorter follow-up periods, traditional surgeries have more extensive long-term data [5]. This gap highlights the need for further research into the long-term efficacy of MIGS and the potential necessity for additional surgical interventions [15].

Cost and accessibility: The cost of MIGS devices and surgical fees can be prohibitive, especially in resource-limited settings. Additionally, the availability of MIGS varies across different regions and healthcare systems [34]. This variation affects the widespread adoption and accessibility of MIGS for all glaucoma patients, posing a challenge to their global implementation.

Challenges in MIGS

Technique-specific learning curve: The challenge lies in the unique mechanisms of action and the specific surgical skills required for each MIGS device, leading to a learning curve for surgeons [21]. To address this, adequate training and experience are crucial. Ongoing education and training programs play a vital role in disseminating these skills among glaucoma surgeons, optimizing surgical outcomes.

Patient selection: Selecting the most suitable candidates for MIGS is a complex task, necessitating consideration of the patient's specific type and severity of glaucoma, as well as overall ocular health [32]. The solution involves comprehensive preoperative evaluation and a tailored approach for each patient, which is essential to maximize the benefits of MIGS.

Integration into treatment paradigms: Integrating MIGS into existing glaucoma treatment paradigms, which include medications, laser therapy, and traditional surgeries, poses a challenge due to the need for a nuanced understanding of their relative benefits and limitations [14]. This challenge can be addressed through continuous education and the development of consensus guidelines, aiding clinicians in making informed decisions about incorporating MIGS into comprehensive treatment plans.

MIGS have undeniably enriched the therapeutic landscape of glaucoma treatment. However, acknowledging their limitations and challenges, as summarized in Table 9, is imperative for their judicious application. As the field of MIGS continues to evolve, addressing these challenges through ongoing research, education, and technological advancement will be key to optimizing patient outcomes in glaucoma management.

**Table 9:** Limitations and Challenges of MIGS Sources: [21,23,27-29,33]

Aspect	Limitation/Challenge	Implication/Addressing Strategy
Efficacy in Severe Glaucoma	Limited efficacy in advanced cases	Suitable primarily for mild to moderate glaucoma
Long-Term Efficacy Data	Lack of extensive long-term data	Need for further research and longer follow-up studies
Cost and Accessibility	High cost and variable availability	Barrier to widespread adoption, particularly in resource-limited settings
Learning Curve	Device and technique-specific skills required	Emphasis on surgeon training and experience
Patient Selection	Critical in determining efficacy	Tailored approach and comprehensive preoperative evaluation
Integration into Paradigms	Balancing with existing treatments	Development of guidelines and continuous education

The future of MIGS

The field of MIGS is dynamic and rapidly evolving, with ongoing research and development of new technologies and techniques. This section highlights recent innovations and research directions that are shaping the future of MIGS, contributing to the continuous improvement of glaucoma management [35].

Recent Innovations in MIGS

Next-generation stents and devices: New stents and devices targeting different aqueous humor outflow pathways represent a significant area of innovation [35]. These advancements include improvements in trabecular bypass technology and novel approaches to uveoscleral and subconjunctival outflow. The implication of these developments is that MIGS are becoming increasingly versatile, offering more tailored approaches for a broader spectrum of glaucoma patients.

Drug-eluting MIGS devices: There is ongoing research into MIGS devices that can both perform surgical interventions and deliver medications directly to the target tissues [36]. This dual functionality aims to enhance IOP control while reducing the dependence on topical medications. The potential of this innovation lies in its ability to increase the efficacy of MIGS and improve patient adherence to treatment plans.

Advanced imaging and diagnostic tools: The incorporation of advanced imaging technologies, such as OCT angiography and real-time intraoperative imaging, is enhancing the precision and safety of MIGS procedures [35,36]. This improved imaging capability allows for more accurate device placement and better assessment of surgical outcomes, which may lead to increased efficacy and fewer complications in MIGS procedures.

Ongoing Research in MIGS

Long-term efficacy and safety studies: Current research is focused on the long-term evaluation of MIGS, aiming to assess their sustained efficacy and safety over extended periods [37]. The expected outcomes of these studies are to provide vital data that will enhance understanding of the long-term benefits and risks associated with MIGS, thereby guiding clinical decision-making.

Comparative effectiveness research: Comparative studies are being conducted between different MIGS devices and also in comparison with traditional surgeries and medical therapies [31]. The outcomes of this research are anticipated to shed light on the relative strengths and limitations of various MIGS options. This will aid in refining the criteria for patient selection, ensuring the most suitable and effective treatments are chosen.

Patient-centered outcome research: There is an increasing emphasis on investigating patient-reported outcomes and quality of life measures following MIGS [32,37]. The insights gained from these studies are expected to guide a more patient-centered approach to glaucoma care. The focus is not only on clinical efficacy but also on patient satisfaction and overall quality of life, providing a more holistic view of treatment outcomes [37].

The future of MIGS is marked by continual innovation and research, driving advancements in glaucoma surgical care. As new devices and techniques emerge, alongside comprehensive research efforts, MIGS are poised to play an increasingly pivotal role in the personalized management of glaucoma, enhancing both clinical outcomes and patient quality of life. This is summarized in Table 10.

**Table 10:** Summary of Future Directions in MIGS Sources: [23-26,35-37]

Innovation/Research Area	Description	Implication for Glaucoma Management
Next-Generation Stents/Devices	New stents targeting diverse outflow pathways	Broadening the applicability of MIGS
Drug-Eluting Devices	MIGS with simultaneous medication delivery	Enhanced efficacy and patient compliance
Advanced Imaging Tools	Improved precision and safety in procedures	Optimized surgical outcomes and reduced complications
Long-Term Efficacy Studies	Extended follow-up on MIGS efficacy and safety	Informed long-term treatment planning
Comparative Effectiveness	Comparisons among MIGS and with other treatments	Refined patient selection and treatment strategies
Patient-Centered Outcomes	Focus on quality of life and patient satisfaction	Holistic approach to patient care in glaucoma

## Conclusions

MIGS have revolutionized glaucoma management by offering a combination of reduced invasiveness, effectiveness, and enhanced safety. These procedures represent a significant shift from traditional, more invasive glaucoma surgeries, providing a balanced option between conservative management and extensive surgeries. Innovations such as the iStent, Hydrus Microstent, XEN Gel Stent, and KDB target various aspects of aqueous humor dynamics, proving especially effective in mild-to-moderate glaucoma cases. Their demonstrated ability to significantly reduce IOP and maintain a favorable safety profile has cemented their role in the field.

Despite their advantages, MIGS face certain challenges, such as limited efficacy in severe glaucoma cases, the need for more long-term data, and issues of cost and accessibility. The learning curve and the necessity for precise patient selection highlight the need for thorough training in MIGS techniques. Nonetheless, these surgeries mark a new chapter in glaucoma care, characterized by continual research and technological advancements, promising improved patient outcomes and a better quality of life for those suffering from this chronic, vision-threatening condition.
